# Abnormal endometrial peristalsis in frozen-thawed embryo transfer: risk factors and improvement of Atosiban treatment

**DOI:** 10.1530/RAF-25-0059

**Published:** 2025-12-11

**Authors:** Yan Su, Yaping Guo, Hui Ji, Mianqiu Zhang, Xiaojing Hou, Xiufeng Ling, Rong Shen

**Affiliations:** ^1^ Nanjing Women and Children’s Healthcare Hospital, Women’s Hospital of Nanjing Medical University, Nanjing, China; ^2^ Nanjing Medical Key Laboratory of Female Fertility Preservation and Restoration, Nanjing, China

**Keywords:** endometrial peristaltic waves, frozen-thawed embryo transfer, clinical pregnancy

## Abstract

**Abstract:**

This retrospective study investigated factors associated with abnormal endometrial peristaltic waves (EPWs) and evaluated the impact of Atosiban intervention on clinical outcomes in frozen-thawed embryo transfer (FET) cycles. A total of 7,554 infertile women undergoing FET cycles were assessed using one-minute transvaginal ultrasound examinations 1 day before transfer to determine peristaltic frequency and direction. Logistic regression identified maternal reproductive history and cycle characteristics as parameters associated with abnormal EPWs. Among women with abnormal EPWs (*n* = 515), Atosiban was administered before embryo transfer, while controls (*n* = 7,039) did not. After propensity score matching (approximately 1:2), 505 treated cycles were compared with 993 control cycles. Overall, pregnancy outcomes were comparable between groups, although Atosiban administration was associated with a modest improvement in clinical pregnancy rate (58.6 vs 53.3%; *P* = 0.049). Subgroup analysis by embryo stage revealed that this benefit was mainly observed in cleavage-stage (day 3) embryo transfers (50.2 vs 41.4%; *P* = 0.025), with no significant effect in blastocyst (day 5/6) transfers. These findings suggest that abnormal EPWs are influenced by multiple clinical factors, and Atosiban intervention may improve pregnancy outcomes, particularly in cleavage-stage embryo transfer cycles.

**Lay summary:**

A successful pregnancy after frozen embryo transfer cycles depends on proper coordination between the embryo and the uterus. In some cases, the uterus shows abnormal movements – called abnormal EPWs – which may make it harder for implantation. In this study, we analyzed medical data from over 7,500 women who had frozen embryo transfers. About 500 of them showed abnormal EPWs during an ultrasound check before embryo transfer. These women received a medication (Atosiban) that helps relax the uterus. We found that abnormal uterine movements can be caused by several factors. After receiving treatment, women with abnormal movements had pregnancy chances similar to those without the problem. The benefit was even greater when embryos were transferred at an early stage (day 3 embryos). This suggests that detecting and treating abnormal uterine movements could improve the chances of pregnancy for some women undergoing IVF, especially those receiving early-stage embryos.

## Introduction

In recent years, frozen embryo transfer (FET) has become the predominant strategy in assisted reproductive technology (ART), with a lower risk of ovarian hyperstimulation (Shi *et al.* 2018) and reduced cycle cancellation rates (Bergenheim *et al.* 2021). However, there are still suboptimal outcomes in FET cycles due to failed implantation. Some studies have shown that endometrial peristaltic waves (EPWs) are associated with implantation (Zhu *et al.* 2014*b*, Kuijsters *et al.* 2017, Sternberg *et al.* 2021).

EPWs are wave-like endometrial activity driven by uterine contractions (Oike *et al.* 1990), which can be detected by ultrasound scanning (Ijland *et al.* 1996). According to the classification system proposed by Ijland *et al.*, EPWs can be divided into five distinct types: fundal-to-cervical, cervical-to-fundal, opposing (simultaneous cervicofundal and fundocervical movements), irregular (with indeterminate direction), and absent waves (Ijland *et al.* 1996). In the natural menstrual cycle, physiological EPWs play essential reproductive roles. They peak around ovulation (MM *et al.* 1997, Zhu *et al.* 2014*b*), directing sperm transport toward the fallopian tubes (Kunz *et al.* 1998, Kunz *et al.* 2006), and subsequently diminish during the luteal phase to create a favorable environment for implantation (Bulletti *et al.* 2000, Kunz & Leyendecker 2002, Oki *et al.* 2002). Physiologic EPWs are essential in reproductive function and embryo implantation (Kunz *et al.* 1997, Kuijsters *et al.* 2017). However, high-frequency EPWs before ET may significantly lower clinical pregnancy rates (CPRs) (Fanchin *et al.* 2001, Zhu *et al.* 2014*a*, Kuijsters *et al.* 2017). Previous studies have demonstrated that an increased frequency of uterine contractions adversely affects implantation outcomes. Specifically, more than three contractions per minute have been associated with reduced implantation rates during embryo transfer (ET) in *in vitro* fertilization (IVF) cycles (Fanchin *et al.* 1998, Zhu *et al.* 2014*a*). Furthermore, a contraction frequency exceeding two per minute at the time of ET has been linked to a nearly 50% reduction in pregnancy rates (OR = 0.52; 95% CI: 0.38–0.69) (Vidal *et al.* 2025).

Numerous studies have investigated the factors of abnormal EPWs and the effect of intervention on FET outcomes. However, the results remain inconsistent, and the necessity of intervention remains controversial. Given the potential impact of abnormal EPWs on implantation, pharmacological interventions to modulate uterine contractility have been explored. Atosiban, the best-known combination of oxytocin and vasopressin V1A receptor antagonist, is uterine-specific and commonly used to halt uterine contractions in patients threatening preterm labor (Tsatsaris *et al.* 2004). For patients with abnormal EPWs, intravenous Atosiban was administered to modulate abnormal uterine contractility (Pierzynski 2011). However, the role of Atosiban in FET cycles remains unclear. In this study, we aimed to identify factors associated with abnormal EPWs and determine which patient subgroups might benefit from targeted Atosiban intervention.

## Methods

### Study design

This retrospective study was conducted from January 2022 to January 2024 at the Women’s Hospital of Nanjing Medical University. A total of 8,202 patients who underwent FET cycles were included. After excluding patients with missing data, 7,554 patients met the inclusion criteria (515 in the abnormal EPWs group and 7,039 in the control group). Among these, in the abnormal group, 248 patients underwent day 3 ET, and 267 patients underwent day 5/6 ET; in the control group, 2,137 patients underwent day 3 transfer, and 4,902 patients underwent day 5/6 transfer. Propensity score matching (PSM) was performed at a 1:2 ratio based on Atosiban application during the peri-implantation stage. The final sample sizes of the abnormal-treated and control groups were 505 and 993, respectively. Ethics approval was obtained (2022KY-161), and data were analyzed anonymously. Informed patient consent was not required.

### Endometrial preparation

Four protocols were used for endometrial preparation in FET: natural cycles (NC-FET), controlled ovarian stimulation cycles (COS-FET), hormone replacement therapy cycles (HRT-FET), and HRT with GnRH agonist pretreatment cycles (GnRHa + HRT-FET).

In NC-FET, ovulation was monitored by transvaginal ultrasound (TVUS). COS-FET was applied in women with inadequate follicular growth, using mild stimulation with letrozole (Fure, Hansoh Pharma, China). HRT-FET was performed in women with irregular menstruation or requiring flexible scheduling. From day 2 or 3 of the menstrual cycle, patients received oral estradiol valerate (Femoston, Abbott Biologicals B.V., the Netherlands) at 4–6 mg/day for 1 week. Endometrial thickness and pattern were then monitored by TVUS, and the estradiol dose was gradually adjusted to 6–12 mg/day, continued until the pregnancy test. In GnRHa + HRT – FET, a single intramuscular injection of triptorelin acetate (Diphereline, Ipsen, France) was administered on day 2 or 3 of the menstrual cycle, followed by the HRT protocol described above.

In all protocols, luteal phase support (LPS) was initiated on the day of ovulation or the first day of progesterone initiation (P + 0). Progesterone was started when estrogen had been given for at least 10 days, and the following criteria were met: serum *E*_2_ ≥ 200 pg/mL, serum *P* ≤ 1.5 ng/mL, and endometrial thickness ≥7 mm. Patients received intramuscular progesterone 40 mg once daily (Xianju Pharma, China) with or without 90 mg of vaginal progesterone (Crinone, Merck Serono, UK) once per day. All cleavage-stage embryos were transferred on P + 3 and blastocysts on P + 5. LPS was continued until the pregnancy test on day 14 after ET. If pregnancy was confirmed, progesterone supplementation was maintained until 10–12 weeks of gestation; if negative, treatment was discontinued.

### Observation and assessment of EPWs

All TVUS examinations were performed using the same diagnostic ultrasound system (ACUSON X300 Premium Edition, model 10566144; Siemens Medical Solutions USA, Inc.) equipped with an endovaginal probe EV9-4 (bandwidth 4–9 MHz). Examinations were conducted by two experienced examiners trained to apply harmonized standards for TVUS scans.

On the morning of the day before ET, patients were instructed to empty their bladder and lie in the lithotomy position. The transvaginal probe was gently inserted and positioned at the uterine mid–sagittal plane to visualize the endometrium. EMT and pattern were recorded. Each observation lasted for one minute. The direction and frequency of EPWs were evaluated. EPWs were classified as abnormal if the frequency exceeded three contractions per minute, if contractions predominantly propagated in the fundal-to-cervical or cervical-to-fundal direction, or if the contraction pattern was irregular. Throughout the examination, participants were asked to relax their abdominal muscles, breathe slowly, and avoid coughing or straining to prevent intra-abdominal pressure changes.

### Atosiban intervention

Patients in the abnormal group received 37.5 mg of Atosiban acetate injection (Atosiban Acetate Injection; Hainan Zhonghe Pharmaceutical Co., Ltd, China) dissolved in 250 mL of normal saline. The intravenous infusion started 30 min before ET and continued for 2 h. The control group received no intervention.

### Pregnancy outcome assessment

Pregnancy outcome was followed up to the first trimester. The primary outcome was CPR. Secondary outcomes included implantation rate (IR), ectopic pregnancy rate (EPR), multiple pregnancy rate (MPR), miscarriage rate (MR), and live birth rate (LBR).

CPR = number of clinical pregnancies/total number of transfer cycles × 100%; IR = number of gestational sacs/total number of embryos transferred × 100%; EPR = number of ectopic pregnancies/total number of clinical pregnancies × 100%; MPR = number of cases with ≥2 gestational sacs detected by ultrasound/number of clinical pregnancies × 100%; MR = number of miscarriages/number of clinical pregnancies × 100%; LBR = number of live births/total number of transfer cycles × 100%.

### Statistical analysis

SPSS 24.0 software (IBM, USA) was used for data analysis. Continuous variables were tested for normality using the Shapiro–Wilk test and were found to be non-normally distributed. Therefore, all continuous variables were analyzed using the Mann–Whitney U test and described as the median (first quartile, third quartile). Categorical variables were expressed as a rate (%) and analyzed using a *χ*2 test. Logistic regression was performed to identify factors influencing abnormal EPWs. A 1:2 PSM was used to adjust for covariates between the abnormal-treated and control groups to reduce baseline differences. A two-sided *P* < 0.05 was considered statistically significant.

## Results

### Factors associated with abnormal EPWs

Abnormal EPWs were observed in 515 patients and were related to duration of infertility, parity (≥1), tubal disease, male factor, and unknown factor infertility (*P* < 0.001). Women with a history of an ectopic pregnancy and uterine anomalies were more likely to have abnormal EPWs (17.9 vs 14.4%, *P* = 0.030; and 6.4 vs 3.5%, *P* = 0.001, respectively), while the opposite was observed in those with a history of cesarean delivery (8.3 vs 12.3%, *P* = 0.007) (Table 1A). In addition, anti-Müllerian hormone was slightly lower in the abnormal group (*P* = 0.047). Cycle characteristics were also compared between the abnormal and control groups. Hormone replacement therapy (HRT) and gonadotropin-releasing hormone agonist (GnRHa) protocols were more prevalent in the abnormal group (*P* < 0.001). Differences were also noted in EMT, luteal transformation time, whether pre-implantation genetic testing (PGT) was performed, and cycle rank (*P* < 0.001) (Table 1B).

**Table 1:** Clinical (A) and cycle (B) characteristics between abnormal and control groups. Data are expressed as mean ± SD for continuous variables following a normal distribution, as median (first quartile, third quartile) for continuous variables not normally distributed, and as *n* (%) for categorical variables.

Characteristics	Abnormal	Control	*P*
	(*n* = 515)	(*n* = 7,039)	
**A) Clinical characteristics**			
Maternal age (years)	32.0 ± 4.7	31.9 ± 5.0	0.591
Paternal age (years)	33.4 ± 5.8	33.2 ± 5.9	0.447
Type of infertility			0.369
Primary	265 (51.5)	3,766 (53.5)	
Secondary	250 (48.5)	3,273 (46.5)	
Duration of infertility (years)	3.9 ± 2.8	3.5 ± 2.7	<0.001
Gravidity (≥1)	263 (51.1)	3,696 (52.5)	0.528
Parity (≥1)	79 (15.3)	1,726 (24.5)	<0.001
Body mass index (kg/m^2^)	22.7 ± 3.5	22.6 ± 3.3	0.904
Cause of infertility, *n* (%)			
Tubal disease	258 (50.1)	4,389 (62.4)	<0.001
Ovulation dysfunction	48 (9.3)	718 (10.2)	0.523
AMA or DOR	61 (11.8)	728 (10.3)	0.282
Endometriosis	27 (5.2)	253 (3.6)	0.056
Male factor	57 (11.1)	432 (6.1)	<0.001
Unknown factor	64 (12.4)	519 (7.4)	<0.001
Previous history, *n* (%)			
Ectopic pregnancy	92 (17.9)	1,011 (14.4)	0.030
Uterine malformation	33 (6.4)	246 (3.5)	0.001
Uterus after septum resection	8	89	
Unicornuate uterus	19	53	
Didelphic uterus	3	10	
Arcuate uterus	3	94	
Cesarean section	43 (8.3)	869 (12.3)	0.007
Intrauterine adhesions	52 (10.1)	763 (10.8)	0.600
Basal hormone levels			
FSH (mIU/mL)	8.1 ± 2.8	8.0 ± 2.9	0.528
LH (mIU/mL)	5.1 ± 3.5	5.2 ± 3.3	0.434
Estradiol (pg/mL)	44.4 ± 22.7	43.6 ± 21.2	0.415
E_2_ on transformation day (pg/mL)	417.7 ± 347.8	421.1 ± 291.2	0.829
AMH (ng/mL)	3.67 (1.84–6.24)	3.96 (2.15–6.65)	0.304
**B) Cycle characteristics**			
Cycle rank	2 (1–3)	1 (1–2)	<0.001
Endometrial thickness (mm)	9.0 (8.0–10.0)	9.0 (8.0–11.0)	0.003
PGT cycle, *n* (%)	41 (8.0)	236 (3.4)	<0.001
Endometrial preparation protocol, *n* (%)			<0.001
Natural cycle	43 (8.3)	1,140 (16.2)	
Controlled ovarian stimulation	33 (6.4)	995 (14.1)	
HRT	280 (54.4)	3,054 (43.4)	
GnRHa + HRT	159 (30.9)	1,850 (26.3)	
Luteal transformation time			<0.001
3 days	248 (48.2)	2,137 (30.4)	
5 days	267 (51.8)	4,902 (69.6)	

AMA, advanced maternal age; DOR, decreased ovarian reserve; FSH, follicle-stimulating hormone; E2, estradiol; AMH, anti-Müllerian hormone; PGT, preimplantation genetic testing; LH, luteinizing hormone; HRT, hormone replacement therapy; GnRha + HRT, gonadotropin-releasing hormone agonist combined with hormone replacement therapy.

**Figure 1:**
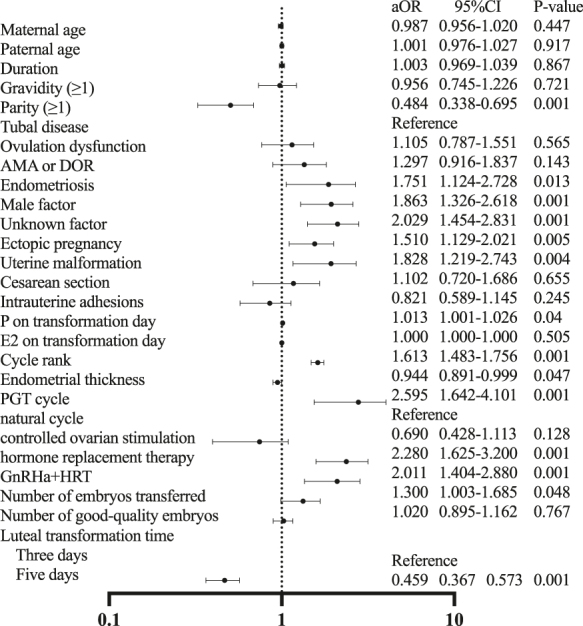
Binary regression analysis. AMA, advanced maternal age; DOR, decreased ovarian reserve; PGT, pre-implantation genetic testing; GnRha + HRT, gonadotropin-releasing hormone agonist combined with hormone replacement therapy; aOR, adjusted odds ratio; CI, confidence interval.

Furthermore, Fig. 1 forest plot showed the results of binary logistic regression analyzing factors associated with abnormal EPWs during the peri-implantation stage.

### Comparison of clinical outcomes after Atosiban intervention

To minimize baseline differences and compare the clinical outcomes, an approximately 1:2 PSM was performed, resulting in a final sample size of 505 in the abnormal-treated group and 993 in the control group. There was no statistically significant difference in baseline characteristics between the two groups (*P* > 0.05; Supplementary Table 1 (see section on Supplementary materials given at the end of the article)). Post-PSM analysis showed that the abnormal-treated group achieved clinical outcomes comparable to those in the control group after the administration of Atosiban (*P* > 0.05), with a modest increase observed in the CPR (58.6 vs 53.3%; *P* = 0.049) and a statistically significant improvement in the live birth rate (LBR) (*P* < 0.001, Table 2).

**Table 2:** Pregnancy outcomes of patients in the abnormal and control groups after propensity score matching.

	Overall	D3 embryo transfer		*P*	D5/6 embryo transfer		*P*
	*P*	Treated	Control		Treated	Control	
		(*n* = 241)	(*n* = 490)		(*n* = 264)	(*n* = 503)	
IR	0.053	148/478 (31.0)	256/980 (26.1)	0.053	220/427 (51.5)	402/807 (49.8)	0.568
CPR	0.049	121/241 (50.2)	203/490 (41.4)	0.025	175/264 (66.3)	326/503 (64.8)	0.683
EPR*	0.784	1/241 (0.4)	6/490 (1.2)	0.436	3/264 (1.1)	4/503 (0.8)	0.697
MPR	0.898	26/121 (21.5)	53/203 (26.1)	0.349	45/175 (25.7)	76/326 (23.3)	0.549
MR	0.303	21/121 (17.4)	43/203 (21.2)	0.403	27/175 (15.4)	58/326 (17.8)	0.502
LBR	<0.001	100/241 (41.5)	160/490 (32.7)	0.019	148/264 (56.1)	268/503 (53.3)	0.463

* Fisher exact test.

IR, implantation rate; CPR, clinical pregnancy rate; EPR, ectopic pregnancy rate; MPR, multiple pregnancy rate; MR, miscarriage rate; LBR, live birth rate. Data are expressed as numbers (percentages) for categorical variables.

To further explore which type of ET benefited more, stratified analysis based on embryonic stage revealed a significant improvement in the CPR and LBR for cleavage-stage embryo transfers in the abnormal-treated group compared to the control group (50.2 vs 41.4%, *P* = 0.025; 41.5 vs32.7%, *P* = 0.019, Table 2). In addition, no statistical differences were found in the baseline characteristics across these subgroups (*P* > 0.05; Supplementary Table 2).

## Discussion

EPWs persist throughout the menstrual cycle as a form of mechanical force (van Gestel *et al.* 2003). Abnormal EPWs have been observed in approximately 30% of patients during IVF-ET cycles (Pierzynski 2011). Our study identified multiple factors associated with abnormal EPWs and suggested that administration of Atosiban may alleviate their detrimental effects on embryo implantation, particularly in cleavage-stage transfers.

Based on the comparison between normal (*n* = 7,039) and abnormal EPWs (*n* = 515) groups and binary logistic regression analysis, several factors were associated with abnormal EPWs. Among these, only parity and a luteal transformation time of 5 days were negatively associated with the abnormal waves (aOR: 0.484, *P* = 0.001; aOR: 0.459, *P* = 0.001), indicating a protective effect. In contrast, all other factors showed a positive association with abnormal EPWs, suggesting an increased risk (aOR > 1). High parity and adequate luteal transformation time may exert protective effects, possibly by optimizing uterine contractility and endometrial receptivity. Some studies have reported an association between parity and uterine contraction frequency (Ryan *et al.* 2019), which may be related to uterine remodeling and adaptation during long-term pregnancy (Paliulyte *et al.* 2017). In FET cycles, the extended endometrial transformation period is accompanied by reduced and more stable uterine peristalsis, which may be associated with improved endometrial receptivity, vascular remodeling, and more complete decidualization (Ramathal *et al.* 2010, Labarta *et al.* 2021).

The remaining factors may cause abnormal EPWs through altered uterine mechanical force transmission, hormonal imbalance, or mental and psychological stress. Excessive uterine peristaltic activities are associated with the pathogenesis of endometriosis (Leyendecker *et al.* 2004, Salmeri *et al.* 2024), and they may also reflect altered fallopian tube function, influence embryo transport, and influence implantation site selection (Pavlova 2019). A study investigated the ET frequency and uterine contractions, revealing that patients exhibited an increased tendency for uterine contractions during the third cycle or beyond (He *et al.* 2016). In our study, abnormal EPWs were more frequently observed in artificial cycles, which may be related to hormonal regulation. Ultrasound examination revealed that EPWs increased at the time of ET (Ayoubi *et al.* 2003), which is in line with evidence that EPWs are positively associated with supraphysiological estradiol levels and oxytocin receptor expression in IVF cycles (Richter *et al.* 2004, Kunz *et al.* 2006). In NC-FET cycles, endogenous progesterone and hCG from the corpus luteum promote uterine relaxation by suppressing gap junction formation and reducing electrical propagation in myometrial cells, which hCG and placental corticotropin-releasing hormone maintain myometrial quiescence (Slattery *et al.* 2001, Tyson *et al.* 2009) via cAMP-dependent pathways (Price & Bernal 2001, Garfield & Maner 2007). In contrast, HRT cycles rely on exogenous hormones that may not accurately mimic natural rhythm and may result in unstable hormonal environments. Furthermore, exogenous estrogen used in HRT protocols may upregulate contractile proteins and oxytocin receptors, further enhancing uterine peristalsis (Garfield & Maner 2007). Elevated ET frequency and infertile duration may also exert substantial pressure, leading to heightened mental stress during transplantation. Such stress can activate the sympathetic nervous system, leading to increased circulating catecholamines. Experimental evidence indicates that catecholamines exert receptor-specific effects on the uterus: epinephrine via β-receptors reduces contractions, whereas norepinephrine via α-receptors enhances them. Accordingly, stress during ET may shift the catecholamine balance toward norepinephrine dominance, leading to enhanced or dysregulated uterine peristalsis, which can disrupt the rhythmic contractions required for optimal embryo implantation (Marcus *et al.* 1996, Segal *et al.* 1998). Moreover, psychological stress may alter endometrial function, potentially contributing to impaired implantation outcomes (Wu *et al.* 2021). However, the specific underlying mechanism has not been established (Kuijsters *et al.* 2017).

Currently, the primary drugs used to inhibit uterine contractions include β-receptor agonists, calcium channel blockers, oxytocin receptor antagonists, and antispasmodics (Zingg & Laporte 2003, Flenady *et al.* 2014, Markiewicz & Jaroszewski 2016). Oxytocin antagonists have been shown to reverse the adverse effects of elevated estradiol and oxytocin on parameters related to endometrial receptivity (Sztachelska *et al.* 2019). Atosiban has demonstrated potential in reducing uterine contractions and improving ART outcomes, and its safety in pre-pregnancy use is well established (Tsatsaris *et al.* 2004, Akerlund 2006, Pierzynski 2011). It is generally well tolerated, with only mild side effects such as headache, nausea, or injection site reactions, and no severe adverse events have been reported in the studies conducted to date (Craciunas *et al.* 2021).

In our study, women in the abnormal-treated groups (*n* = 505) achieved comparable clinical outcomes to those of the control group (*n* = 993). The CPR in the intervention group was even slightly higher than that in the control group (58.6 vs 53.3%, *P* = 0.049). However, the LBR in the intervention group remained significantly lower than in the control group (*P* < 0.001). The effectiveness of Atosiban is still controversial. A randomized, double-anonymized, controlled trial conducted by Tang *et al.* (2022) showed that using Atosiban before FET may not improve the LBR of RIF patients. A systematic review and meta-analysis of interventions to optimize ET in assisted reproduction concluded that using Atosiban during transfer may improve clinical and ongoing pregnancy rates (Schwarze *et al.* 2020, Tyler *et al.* 2022). In a meta-analysis evaluating the effect of Atosiban on pregnancy outcomes, involving 1,754 women undergoing IVF, Atosiban administration was linked to a higher IR and CPR; however, no significant difference was observed in LBR (Huang *et al.* 2017).

To further explore which type of ET benefited more, subgroup analysis by embryonic stage of ET showed that the therapeutic intervention provided benefits in both day 3 (*n* = 731) and day 5/6 (*n* = 767) embryo transfers. The greatest benefit of Atosiban intervention (*n* = 241) was observed in patients undergoing cleavage-stage ET, with a significant improvement in CPR and LBR compared with controls (*n* = 490) (50.2 vs 41.4%, *P* = 0.025; 41.5 vs 32.7%, *P* = 0.019). In contrast, no significant benefit was observed in blastocyst (day 5/6) transfers (66.3 vs 64.8%, *P* = 0.683; 56.1 vs 53.3%, *P* = 0.463). Embryos in the blastocyst stage are about to hatch from the zona pellucida or have already hatched, leading to rapid invasion and implantation after transplantation. During the cleavage stage, the embryo will continue to grow and migrate within the uterine cavity; abnormal peristalsis may expel the seed from the uterine cavity and impair implantation. Consequently, day 3 embryos could benefit more from Atosiban treatment. However, implantation is a multifactorial process, and other uterine or embryo-related factors may also influence the differential outcomes observed between cleavage-stage and blastocyst transfers.

Our study investigated factors associated with abnormal EPWs. By identifying potential influencing factors, this study provides clinical insight into the recognition of high-risk populations and the potential benefits of timely therapeutic intervention. However, the findings may not be generalizable to populations with different ethnic backgrounds, IVF protocols, or comorbidities because of their limitations. First, it was a single-center retrospective study, which may induce selection bias. Second, although PSM analysis was performed for known confounders, embryo implantation is influenced by multiple factors, and residual confounding from unmeasured factors such as lifestyle, mental state, or genetic background cannot be excluded. Third, only Atosiban was used as the sole intervention in the abnormal group, and a control group with abnormal EPWs who did not receive medication was lacking.

Abnormal EPWs during FET cycles are influenced by multiple factors, and peri-implantation intervention with Atosiban appears to improve pregnancy outcomes for cleavage embryo transfers. In the future, well-designed prospective randomized controlled trials are warranted to validate and extend these findings. Moreover, further studies could stratify patients with abnormal EPWs into specific subgroups according to their risk factors to better elucidate the underlying mechanisms and optimize individualized treatment strategies.

## Supplementary materials



## Declaration of interest

The authors declare that there is no conflict of interest that could be perceived as prejudicing the impartiality of the work reported.

## Funding

This work was funded by grants from the National Natural Science Foundation of Chinahttps://doi.org/10.13039/501100001809 (Grant No. 82071648).

## Author contribution statement

YS, YG, and HJ contributed to the conceptualization and methodology of the study. YG and YS were responsible for drafting the manuscript. MZ and XH conducted the transvaginal ultrasound examinations, participated in data acquisition, and contributed to the review and editing of the manuscript. XL and RS provided supervision, project administration, and final validation of the study. All authors have read and approved the final version of the manuscript for publication.

## Data availability

All the data are presented in the paper and the supplementary materials. Additional data are available from the corresponding author upon request.

## Ethics approval and consent to participate

The study was conducted according to the guidelines of the Declaration of Helsinki and approved by the ethics committee of the Women’s Hospital of Nanjing Medical University in 2022 (2022KY-161). Informed patient consent was not required.
