# Supplementary material for: Abnormal endometrial peristalsis in frozen-thawed embryo transfer: risk factors and improvement of Atosiban treatment
Source: Reprod Fertil. 2025 Dec 11;6(4):e250059. doi: 10.1530/RAF-25-0059 (PMC12701712; doi:10.1530/RAF-25-0059)
Supplement: Supplementary file 1 [file supplementary_materials.pdf]

Supplemental Table1. Clinical and cycle characteristics between Abnormal and control groups after PSM

Items	Abnormal-treated (n=505)	Control (n=993)	*P*
Maternal age (years)[a]	31.9 ± 4.7	32.0 ± 5.0	0.743
Paternal age (years)[a]	33.3 ± 5.8	33.6 ± 5.9	0.298
Type of infertility[c]			0.175
Primary	259 (51.3)	546 (55.0)	-
Secondary	246 (48.7)	447 (45.0)	-
Infertile duration (years)a	3.8 ± 2.7	4.0 ± 3.0	0.408
Gravidity (≥1) [c]	256 (50.7)	537 (54.1)	0.215
Parity (≥1) [c]	79 (15.6)	166 (16.7)	0.595
Body mass index[a]	22.7 ± 3.5	22.6 ± 3.3	0.874
Cause of infertility, n (%) [c]			0.812
Tubal disease	256 (50.7)	543 (54.7)	-
Ovulation dysfunction	48 (9.5)	83 (8.4)	-
AMA or DOR	60 (11.9)	121 (12.2)	-
Endometriosis	27 (5.3)	51 (5.1)	-
Male factor	53 (10.5)	92 (9.3)	-
Unknown factor	34 (6.7)	58 (5.8)	-
Others	27 (5.3)	45 (4.5)	-
Previous history, n (%) [c]			
Ectopic pregnancy	88 (17.4)	202 (20.3)	0.177
Uterine malformation	31 (6.1)	63 (6.3)	0.877
uterus after septum resection	8	24	-
unicornuate uterus	18	3	-
didelphic uterus	2	3	-
arcuate uterus	3	24	-
Cesarean section	39 (7.7)	72 (7.3)	0.742
Intrauterine adhesions	49 (9.7)	84 (8.5)	0.424
Hormone levels			
Basal FSH (mIU/mL)	8.1 ± 2.8	8.2 ± 3.0	0.792
Basal LH (mIU/mL)	5.1 ± 3.6	5.1 ± 2.9	0.627
Basal E_2 (pg/mL)	44.6 ± 22.8	44.4 ± 21.9	0.894
E_2 on P_4 administration day (pg/mL)	417.4 ± 287.0	418.1± 318.4	0.964
AMH (ng/mL) [b]	3.7(1.9-6.4)	3.7(2.0-6.3)	0.601
Cycle rank[b]	2(1-3)	2(1-3)	0.691
Endometrial thickness (mm)[a]	9.3 ± 1.7	9.3 ± 1.8	0.993
PGT cycle, n (%) [c]	39 (7.7)	62 (6.2)	0.280
EPP, n (%) [c]			0.740
NC	43 (8.5)	94 (9.5)	-
COS	32 (6.3)	68 (6.8)	-

HRT	276 (54.7)	553 (55.7)	-
GnRHa+HRT	154 (30.5)	278 (28.0)	-
Number of transferred embryos[b]	2(2-2)	2(2-2)	0.761
Good quality embryos[b]	1(0-1)	1(0-1)	0.631
Luteal transformation time[c]			0.553
three days	241 (47.7)	490 (49.3)	-
five or six days	264 (52.3)	503 (50.7)	-

a: Data are expressed as the mean ± SD for continuous variables following a normal distribution. b: Data are expressed as the median (first quartile, third quartile) for continuous variables not normally distributed. c: Data are expressed as numbers (percentages) for categorical variables. GnRha+HRT: gonadotropin-releasing hormone agonist combined with hormone replacement therapy; AMA: advanced maternal age; DOR: decreased ovarian reserve; GnRha+HRT: gonadotropin-releasing hormone agonist combined with hormone replacement therapy

Supplemental Table2. Clinical and cycle characteristics between abnormal-treated and control groups of different embryonic stages

Items	D3 embryo transfer		*P*	D5/6 embryo transfer		*P*
	Treated (n=241)	Control (n=490)		Treated (n=264)	Control (n=503)	
Maternal age (years) [a]	33.0 (29.0-36.0)	33.0 (29.0-36.0)	0.718	31.0 (28.0-33.0)	31.0 (28.0-33.0)	0.782
Paternal age (years) [a]	33.0 (30.0-37.0)	32.5 (30.0-38.0)	0.651	31.0 (29.0-35.0)	32.0 (29.0-36.0)	0.404
Type of infertility [b]			0.470			0.232
Primary	124 (51.5)	266 (54.3)	-	135 (51.1)	280 (55.7)	-
Secondary	117 (48.5)	224 (45.7)	-	129 (48.9)	223 (44.3)	-
Inf.duration (years) [a]	4.0 (2.0-6.0)	3.0 (2.0-5.0)	0.418	3.0 (2.0-5.0)	3.0 (2.0-5.0)	0.316
Gravidity (≥1) [b]	123 (51.0)	264 (53.9)	0.470	133 (50.4)	273 (54.3)	0.304
Parity（≥1） [b]	41 (17.0)	94 (19.2)	0.477	38 (14.4)	72 (14.3)	0.976
Body mass index [a]	22.1 (20.3-24.8)	22.4 (20.3-24.8)	0.533	22.2 (20.0-24.7)	21.7 (20.0-24.4)	0.411
COI n (%) [b]			0.124			0.963
Tubal disease	119 (49.4)	272 (55.5)	-	137 (51.9)	271 (53.9)	-
Ovulation dysfunction	16 (6.6)	32 (6.5)	-	32 (12.1)	51 (10.1)	-
AMA or DOR	45 (18.7)	91 (18.6)	-	15 (5.7)	30 (6.0)	-
Endometriosis	16(6.6)	31 (6.3)	-	11 (4.2)	20 (4.0)	-
Male factor	28 (11.6)	38 (7.8)	-	25 (9.5)	54 (10.7)	-
Unknown factor	14 (5.8)	26 (5.3)	-	20 (7.6)	32 (6.4)	-
Others	3(1.2)	0 (0)	-	24 (9.1)	45 (8.9)	-
Previous history n (%) [b]						
Ectopic pregnancy	37 (15.4)	92 (18.8)	0.254	51 (19.3)	110 (21.9)	0.410
Uterine malformation	8 (3.3)	23 (4.7)	0.386	23 (8.7)	40 (8.0)	0.716
Cesarean section	20 (8.3)	41 (8.4)	0.975	19 (7.2)	31 (6.2)	0.582
Intrauterine adhesions	24 (10.0)	35 (7.1)	0.189	25 (9.5)	49 (9.7)	0.904
Basal hormone levels [a]						
FSH (mIU/mL)	8.0 (6.6-9.8)	8.0 (6.7-9.8)	0.925	7.3 (6.2-8.5)	7.3 (6.1-8.5)	0.878
LH (mIU/mL)	4.1 (3.1-5.8)	4.1 (3.0-5.7)	0.965	4.4 (3.3-6.3)	4.6 (3.5-6.4)	0.437
E_2 (pg/mL)	40.0 (29.0-54.1)	42.0 (29.0-54.0)	0.918	40.0 (29.2-52.0)	41.0 (29.0-54.0)	0.761
E2/P4D	338 (266-457)	340 (271-469)	0.366	347 (271-480)	340 (261-449)	0.367
AMH (ng/mL) [a]	3.0 (1.1-5.0)	2.7 (1.3-5.1)	0.981	4.3 (2.6-7.4)	4.6 (2.8-7.5)	0.607
Cycle rank [a]	2 (1-3)	2 (1-3)	0.349	2 (1-3)	2 (1-3)	0.784
EMT (mm) [a]	9.0 (8.0-10.0)	9.0 (8.0-11.0)	0.993	9.0 (8.0-10.0)	9.0 (8.0-10.0)	0.809
EPP, n (%) [b]			0.795			0.118
Natural cycle	24 (10.0)	45 (9.2)	-	19 (7.2)	49 (9.7)	-
COS	11 (4.6)	25 (5.1)	-	21 (8.0)	43 (8.5)	-
HRT	140 (58.1)	270 (55.1)	-	136 (51.5)	283 (56.3)	-
GnRHa+HRT	66 (27.4)	150 (30.6)	-	88 (33.3)	128 (25.4)	-

No. of ET embryos [a]	2 (2-2)	2 (2-2)	0.484	2 (1-2)	2 (1-2)	0.710
Good quality embryos [a]	0 (0-1)	0 (0-1)	0.943	1 (0-2)	1 (0-1)	0.804

a: Data are expressed as the median (first quartile, third quartile) for continuous variables not normally distributed.

b: Data are expressed as percentages (numbers) for categorical variables. Inf.duration: infertile duration; COI: Cause of infertility; AMA: advanced maternal age; DOR: decreased ovarian reserve; FSH: follicle-stimulating hormone; LH: luteinizing hormone; E_2: estradiol; E2/P4D: estradiol on the day of progesterone administration; AMH: anti-Mullerian hormone; EMT: Endometrial thickness; EPP: endometrial preparation protocol; COS: controlled ovarian stimulation; HRT: hormone replacement therapy; GnRha+HRT: Gonadotropin-releasing hormone agonist combined with hormone replacement therapy; No.of ET embryos: numbers of embryos transferred.
